# Awareness regarding risk factors of type 2 diabetes among individuals attending a tertiary-care hospital in Bangladesh: a cross-sectional study

**DOI:** 10.1186/1756-0500-7-599

**Published:** 2014-09-03

**Authors:** Shirin Jahan Mumu, Farzana Saleh, Ferdous Ara, Md Rabiul Haque, Liaquat Ali

**Affiliations:** Department of Epidemiology, Bangladesh University of Health Sciences (BUHS), 125/1 Darussalam, Mirpur, Dhaka 1216 Bangladesh; Department of Community Nutrition, BUHS, Dhaka, Bangladesh; Department of Population Sciences, University of Dhaka, Dhaka, Bangladesh; Department of Biochemistry and Cell Biology, BUHS, Dhaka, Bangladesh

**Keywords:** Diabetes risk factor, Awareness, Knowledge, Attitude, Bangladesh

## Abstract

**Background:**

Awareness regarding risk factors is a prerequisite for the prevention of diabetes in general population. However, there are great variations in the level of this awareness from population to population and this needs to be explored in different ethnic and social groups for designing appropriate preventive strategies. The purpose of the study was to assess the level of awareness regarding the risk factors responsible for the development of type 2 diabetes and its determinants among individuals who attended a tertiary care hospital.

**Methods:**

Under an analytical cross-sectional design, 400 non-diabetic respondents, aged >30 years, were conveniently selected from the Out-Patient Department of BIRDEM, the tertiary care hospital of the Diabetic Association of Bangladesh. A pretested, semi-structured questionnaire was developed to assess knowledge and attitude of the respondents. Respondents’ level of knowledge and attitude were categorized as good, average and poor (GAP). Multivariate along with bivariate statistics was used to measure knowledge and attitude of type 2 diabetes.

**Results:**

Among the respondents the levels of knowledge and attitude were 13%, 10% good; 68%, 75% average and 19%, 14% poor respectively. In multivariate analysis, high literacy (p = 0.0001), respondents who are in service (p = 0.02) and family history of diabetes (p = 0.02) were found significantly associated with the knowledge score after adjustment. Respondents who had passed secondary and higher secondary education had a significant association (p = 0.03) with the attitude score. Housewives had a significantly lower attitude score than others (p = 0.04). Family history of diabetes and knowledge on the risk factors of diabetes showed significant positive association with the attitude score (p = 0.013 and p = 0.0001 respectively).

**Conclusions:**

Overall, respondents participating in this study have average awareness regarding risk factors of diabetes. From a public health perspective, there is a decisive need of innovative prevention programs for targeting people including high-risk individuals.

## Background

Diabetes is now recognized as a major chronic public health problem throughout the world and affecting a large number of people in a wide range of ethnic and economic levels in both developed and developing countries. However, it is estimated that the developing countries will bear the brunt of this epidemic in the 21st century, with 80% of all new cases of diabetes expected to appear in the developing countries by 2025 [1] including South Asian countries like Bangladesh [2]. The International Diabetes Federation (IDF) estimates, Bangladesh has nearly 8.4 million of diabetes patients which is expected to reach to 16.8 million by 2030 [3]. In Bangladesh most of the patients are type 2 diabetics and the risk of developing type 2 diabetes mellitus (DM) is determined by some modifiable factors related to rapid urban growth and changing lifestyle (i.e. obesity, sedentary lifestyle, diet, smoking, physical and emotional stress) and non-modifiable factors (i.e. family history of diabetes, age, race/ethnicity) [4, 5]. The rising prevalence of type 2 diabetes in Bangladesh is primarily attributed to rapid urbanization and associated changes in lifestyle, such as sedentary lifestyle, higher-calorie food intake, and stressful life. However, evidence suggests that lifestyle related interventions targeting modifiable risk factors can either prevent or delay the onset of type 2 diabetes [5–7].

Prevention of diabetes is important because it is costly both in human and monetary matters [8]. Awareness of risk factors is a prerequisite to prevent diabetes among general population and also in high-risk groups, such as Impaired Fasting Glucose (IFG) and Impaired Glucose Tolerance (IGT). If people are aware of the risk factors that develop diabetes, the rate of its occurrence can be minimized**.** Evidence eventually reported that people who perceive themselves to be at risk of a disease are more likely to engage in and comply with efforts to reduce their risk of developing the problem [9–11]. Thus, considerable efforts are needed to inform people about diabetes [12] to judge their risk including the severity and probability of ill effects, about the risk factors that modify their susceptibility, as well as the ease or difficulty of avoiding harm [13]. Acquiring knowledge on the level of awareness among population about diabetes is the first step in formulating a prevention program for diabetes. Such data are extremely important to plan public-health polices with specific reference to implementation of national diabetes control programs [14]. Additionally, there are great variations in the level of this awareness from population to population, and this needs to be explored in different ethnic and social groups for designing appropriate preventive strategies.

Only a few studies have conducted on knowledge regarding and attitude towards diabetes, obesity and dyslipidemia among Bangladeshi diabetic patients [15–17]. However, the level of awareness regarding risk factors in development of diabetes among the Bangladeshi population gets very little attention in previous study. This study was therefore, conducted to assess the level of awareness about the risk factors responsible for the development of type 2 diabetes and its determinants among individuals who attended a tertiary care centre.

## Methods

Under an analytical cross-sectional design, 400 non-diabetic respondents, aged >30 years, were conveniently selected from the Out-Patient Department of BIRDEM, the tertiary care hospital of the Diabetic Association of Bangladesh. The reason behind selecting >30 years non diabetic individuals is that the risk of type 2 diabetes is likely to increase at age ≥ 40. Assessing the knowledge and attitudes regarding the contributing risk factors for type 2 diabetes of non-diabetic people in early age is important for primary prevention. Respondents who had a previous record of glucose intolerance or other medical complications and who were hospitalized and unable to respond to a short list of simple questions were excluded from the study. A semi-structured questionnaire was developed in local language (Bangla) and feedbacks were adjusted after pretest. The questionnaire was divided into two parts. The first part contained socio-demographic information, family history of diabetes, and sources of information on diabetes. The second part included 12 questions to assess knowledge and 9 attitude statements regarding the risk factors of diabetes.

The purpose of obtaining data was to assess the level of knowledge possessed by the respondents about and attitudes towards the risk factors of diabetes.. The knowledge scale required the respondents to rate each item as either “correct”, “incorrect”, or “don’t know”. For each response, the respondents were also requested to note from where they got the information. For the knowledge questions, 1 mark was assigned for each correct answer and 0 for incorrect answer. For each respondent, a sum of score was calculated to define a knowledge variable. The respondents could score between 0 and 12. From this study it was found that the mean [±standard deviation (SD)] knowledge score for the respondents was 6 ± 2. During analyses the level of knowledge was classified according to the score - poor knowledge corresponded to score <4 (i.e. < mean – 1 SD); average knowledge corresponded to score between 4 and 8 (i.e. mean ± 1 SD) and good knowledge corresponded to score of >8 (i.e. > mean + 1 SD) [18, 19]. Additionally, a three-point Likert scale was used for assessing the attitudes of the respondents [20]. The minimum possible score was 3, and the maximum possible score was 27. The attitude of the respondents was also classified according to each respondent’s score while analyses. The mean ± standard deviation (SD) attitude score was 23 ± 3. Poor attitude corresponded to a score of <20 (i.e. < mean - 1 SD); average attitude corresponded to a score between 20 and 26 (i.e. mean ± 1 SD); and good knowledge corresponded to a score of >26 (i.e. >mean + 1 SD) [18, 19]. Body weight was measured on a lever balance (Detecto-Medic, Detecto Scales, Inc, USA) and height was measured barefooted in the standing position with a standard scale to the nearest 0.1 cm. The body mass index (BMI) criteria for the Asian population were used for identifying overweight and obese of the respondents [21].

Descriptive statistics, univariate and bivariate analyses, and multivariate linear regression were performed using the SPSS software (version 11) (SPSS Inc., Chicago, IL). The statistical tests were considered significant at a level of ≤5% (≤0.05). Bivariate analyses using Spearman**’**s correlation, Pearson’s correlation, Student’s *t*-test, and one-way analysis of variance (ANOVA) were used for assessing the correlations or associations between the knowledge and attitude scores and the continuous or categorical covariates respectively. Multiple linear regressions were performed to determine which determinants were more important as predictors of the respondents’ knowledge and attitude. The dependent variables for each proposed model were knowledge score and attitude score.

Informed written consent was taken from all the respondents after fully explaining the nature and purpose of, and all procedures used for, the study. Ethical approval was obtained from the ethics and research review committees of the Diabetic Association of Bangladesh.

## Results

Table 1 summarizes the characteristics of the study population. Four hundred respondents were enrolled in the study. The mean (±SD) age of the study respondents was 41.86 ± 10.03 years and more than half of them (56%) were female. Of the respondents, 21% were illiterate, and 42% had completed high school education with monthly income, $ median (range), 142.86(28.57-3571). Around half of the respondents (51%) had family history of type 2 diabetes. The mean (±SD) BMI of the respondents was 24.40 ± 3.82, and 43.8% were overweight and 19.3% were obese. The mean knowledge and attitude score of the study respondents were 5.64 ± 2.45 and 22.92 ± 2.93 respectively.

**Table 1:** **Characteristics of the study respondents**

Variables	Descriptive measure
**Age, years,** mean ± SD	41.86 ± 10.03
**Monthly Income , $,** median (range)	142.86 (28.57-3571)
**BMI, kg/m** ^**2**^ **,** mean ± SD	24.40 ± 3.82
**Knowledge score,** mean ± SD	5.64 ± 2.45
**Attitude score,** mean ± SD	22.92 ± 2.93
**Gender**	
Male	176 (44%)
Female	224 (56%)
**Education**	
Illiterate	84 (21%)
Primary to 8^th^ grade	71 (18%)
Secondary-higher secondary	168 (42%)
Graduate & above	77 (19%)
**Occupation**	
Service	101 (25.3%)
Business	69 (17.3%)
Housewife	201 (50.3%)
Unemployed	27 (7.3%)
**Family history of diabetes**	
Have family history	205 (51%)
No family history	195 (48%)
**Acquisition of information regarding diabetes**	
Received information	352 (88%)
Not received information	48 (12%)
**BMI (kg/m** ^**2**^ **)**	
Underweight (<18.5)	13 (3.3%)
Normal (18.5-22.9)	135 (33.8%)
Overweight (23–27.5)	175 (43.8%)
Obese (>27.5)	77 (19.3%)

*BMI* = Body Mass Index.

Descriptive measures are expressed as number (%), mean ± SD and median (range).

**Figure 1:**
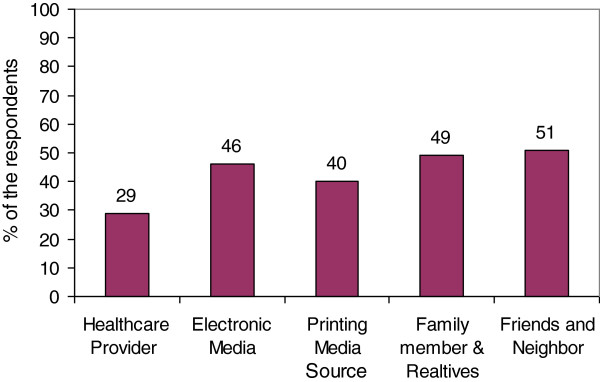
**Sources of information regarding diabetes.**

**Figure 2:**
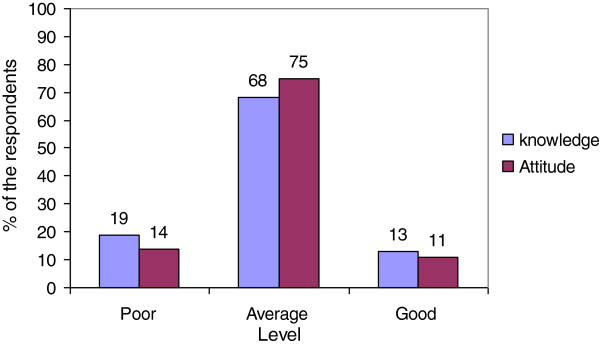
**Level of knowledge and attitude regarding diabetes risk factors.**

Most of the respondents (88%) received information on diabetes from friends, neighbors, relatives, print and electronic media, and healthcare providers. The two most important sources were friends & neighbor (51%) and family (49%) respectively, followed by electronic media (46%) (Table 1 and Figure 1). Figure 2 describes that approximately 68% of respondents had average knowledge regarding the risk factors of diabetes. About 18% had poor knowledge regarding risk factors of T2DM, and 13% had good knowledge. The large majority (75%) of the respondents had average attitude, and 14% had poor attitude. Only 10% had good attitude towards T2DM.

Table 2 illustrates the responses of the respondents to each item in the knowledge question. A substantial number of the respondents were aware of the common risk factors of diabetes, family history of diabetes (55%), increasing age (69%), overweight (71%), lack of regular exercise (73%), mental stress (75%), sedentary lifestyle (81%), and fast food (60%). However, around 20% had no knowledge on these risk factors. Besides, there was a lack of awareness of smoking (65%), gestational diabetes (71%), low birth weight (84%), impaired glucose tolerance (97%), high cholesterol level (67%), and hypertension (55%) as risk factors contributing to the development of diabetes. In case of attitude statement, about 37% said that regular exercise needs a lot of effort and 13% stated that if there is a possibility to occur diabetes, nothing could minimize the risk of it (Table 3).

**Table 2:** **Knowledge regarding risk factors of diabetes among the respondents**

Risk factors	Correct answer (n)%	Incorrect answer (n)%	Don’t know (n)%
Family history	220 (55%)	95 (24%)	85 (21%)
Increasing age	275 (69%)	45 (11%)	80 (20%)
Smoking	111 (28%)	27 (7%)	262 (65%)
Overweight	286 (71%)	29 (7%)	78 (18%)
Lack of regular exercise	293 (73%)	17 (4%)	90 (23%)
Gestational diabetes	102 (26%)	13 (3%)	285 (71%)
Low birth weight	53 (13%)	13 (3%)	334 (84%)
Mental stress	300 (75%)	23 (6%)	76 (19%)
IGT	10 (3%)	0 (0%)	390 (97%)
Sedentary lifestyle	322 (81%)	12 (3%)	66 (16%)
High cholesterol level	122 (31%)	10 (2%)	268 (67%)
Hypertension	153 (38%)	29 (7%)	218 (55%)
Oily foods	274 (69%)	26 (6%)	100 (25%)
Fast food	242 (60%)	19 (5%)	139 (35%)
Soft drinks	258 (65%)	17 (4%)	125 (31%)

Results are expressed as number (%).

IGT = Impaired Glucose Tolerance.

**Table 3:** **Attitude towards diabetes risk factors among the respondents**

Attitude statements	Positive	Undecided	Negative
	n (%)	n (%)	n (%)
It is possible to prevent diabetes by dietary management	335 (83.8)	50 (12.5)	15 (3.8)
Family history of diabetes influences to follow disciplined life	38 (9.5)	166 (41.5)	196 (49)
Obesity increases the risk of diabetes	280 (70)	95 (23.8)	25 (6.3)
Regular exercise needs lots of effort	148 (37)	52 (13)	200 (50)
Everybody should be aware regarding prevention of diabetes with increasing age	298 (74.5)	97 (24.3)	5 (1.3)
If you have possibility to occur diabetes then nothing to do	52 (13)	60 (15)	288 (72)
If you try you can reduce the risk of diabetes	317 (79.3)	70 (17.5)	13 (3.3)
People who take steps to reduce the risk factors of diabetes, their possibility of occurring diabetes is less	297 (74.3)	87 (21.8)	16 (4)
You have little control on your health risk	113 (28.3)	105 (26.3)	182 (45.5)

Results are expressed as number (%).

Item 1, 3, 5, 7 were scored with 3 points for positive, through to 1 point for negative. Item 2, 4, 6, 8, 9 were scored in reverse.

In bivariate analysis, knowledge of respondents was found significantly associated with their attitude (p = 0.0001). The mean knowledge score of male and female was 6.27 ± 2.32 and 5.14 ± 2.45 and this difference was statistically significant (p = 0.0001). Highly significant difference were also found between the scores of four education groups (p = 0.0001). The mean (±SD) knowledge score of illiterate group was 4.06 ± 2.36, whereas graduate group scored 6.14 ± 2.20. The mean knowledge score was significantly lower in housewife and unemployed respondents than the respondents who were in service and business (p = 0.0001). Respondents who had family history of diabetes scored higher than that of who had no family history (6.19 ± 2.33 vs 5.06 ± 2.45; p = 0.0001). Respondents who did not received any information regarding diabetes scored lower than the respondents who get information (4.51 ± 2.30 vs 5.79 ± 2.44; p = 0.001). Higher BMI showed higher knowledge score and the difference of scores among groups are significant (p = 0.04). Significant correlation was found between knowledge score and income (r = 0.263, p = 0.0001) & BMI (r = 0.15, p = 0.003) (Table 4).

**Table 4:** **Bivariate associations of all variables with knowledge and attitude score**

Variables	Association with knowledge score		Association with attitude score	
	Correlation coefficient	***P***value	Correlation coefficient	***P***value
**Age, years,** mean ± SD	0.092	0.067*	0.029	0.56*
**Monthly income , $,** median (range)	0.263	0.0001^†^	0.28	0.0001^†^
**BMI, kg/m** ^**2**^ **,** mean ± SD	0.15	0.003*	0.09	0.07*
**Knowledge score,** mean ± SD	-	-	0.51	0.0001*
**Attitude score,** mean ± SD	0.51	0.0001*	-	-
	**Knowledge score mean ± SD**		**Attitude score mean ± SD**	
**Gender**				
Male	6.27 ± 2.32	0.0001^‡^	23.57 ± 2.87	0.0001^‡^
Female	5.14 ± 2.45		22.41 ± 2.88	
**Education**				
Illiterate	4.06 ± 2.36	0.0001^‡^	21.17 ± 2.59	0.0001^‡^
Primary to 8^th^ grade	4.70 ± 2.14		22.37 ± 2.87	
Secondary-higher secondary	6.14 ± 2.20		23.45 ± 2.66	
Graduate & above	7.14 ± 2.05		24.18 ± 2.96	
**Occupation**				
Service	6.74 ± 2.33	0.0001^§^	23.72 ± 2.99	0.0001^§^
Business	6.10 ± 2.02		23.79 ± 2.40	
Housewife	5.0 ± 2.45		22.17 ± 2.86	
Unemployed	5.17 ± 2.42		23.20 ± 3.14	
**Family history of diabetes**				
Have family history	6.19 ± 2.33	0.0001^‡^	23 ± 3.04	0.79^‡^
No family history	5.06 ± 2.45		23.1 ± 2.74	
**Acquisition of information regarding diabetes**				
Received information	5.79 ± 2.44	0.001^‡^	23.09 ± 2.98	0.0001^‡^
Not received information	4.51 ± 2.30		21.59 ± 2.14	
**BMI (kg/m** ^**2**^ **)**				
Underweight (<18.5)	4.0 ± 2.61	0.007^§^	20.76 ± 3.03	0.035^§^
Normal (18.5-22.9)	5.37 ± 2.28		22.89 ± 2.79	
Overweight (23–27.5)	5.70 ± 2.56		22.92 ± 3.16	
Obese (>27.5)	6.24 ± 2.32		23.33 ± 2.47	

*BMI* = Body Mass Index.

^*^Pearson correlation was used for analysis of continuous, normally distributed variables.

^†^Spearman correlation was used for analysis of continuous, non-normally distributed variables.

^‡^Student *t* test was used to compare mean across normally distributed variables with 2 categories.

^§^One-way ANOVA was used to compare mean and median scores and values across categorical variables with more than 2 categories.

The mean attitude score of male (23.57 ± 2.87) was significantly higher than the mean attitude score of female (22.41 ± 2.88; p = 0.0001). The average attitude score of the illiterate group was also significantly lower than the attitude level of other groups (p = 0.0001). The attitude score was significantly lower in housewife respondents than the respondents of other occupation. Respondents who get information regarding diabetes scored significantly higher than the group who did not get any information (23.09 ± 2.98 vs 21.59 ± 2.14; p = 0.0001). It is surprisingly noted that obese respondents obtained significantly higher score than other groups (p = 0.035). Significant correlation was found between monthly income and attitude score (r = 0.281, p = 0.0001) (Table 4).

In an attempt to identify the factors that might predict the respondents’ probability of having good knowledge and attitude, multivariate linear regression analyses were performed. Variables which were shown significant association with knowledge and attitude score in bivariate analysis were put in the model, though all variables did not show significant in the multivariate analysis. The overall multiple regression model that was used to assess predictions of diabetes risk factor knowledge achieved on R^2^ of 0.27; p = 0.0001. The results showed that high literacy was significantly associated with the knowledge score after adjustment (p = 0.0001). Respondents who were in service had significantly (p = 0.02) higher knowledge score than other occupations. The family history of diabetes was significantly associated with the knowledge score (p = 0.002). Regression analysis also identified significant predictors of respondents’ attitude (R^2^ = 0.31; p = 0.0001). Respondents who had passed secondary and higher secondary education had a significant association (p = 0.03) with the attitude score. Housewives had a significantly lower attitude score than others (p = 0.04). Family history of diabetes and knowledge on the risk factors of diabetes showed significant positive association with the attitude score (p = 0.013 and p = 0.0001, respectively) (Table 5).

**Table 5:** **Multivariable regression analysis of knowledge and attitude score as a dependent variable with other parameters of the respondents**

a. Dependent variable: Knowledge				
Predictor variable	B ^1^ ± SE	Beta ^2^	***P***value	CI for B
Age	0.03 ± 0.92	0.104	0.153	-0.495, 3.137
Sex	-0.101 ± 0.495	-0.02	0.838	-1.074, 0.871
Education				
Illiterate	Reference category	-	-	-
Primary	0.335 ± 0.357	0.052	0.349	-0.367, 1.038
Secondary-higher secondary	1.678 ± 0.32	0.337	0.0001	1.049, 2.306
Graduate and above	2.318 ± 0.426	0.372	0.0001	1.479, 3.156
Occupation				
Unemployed	Reference category	-	-	-
Housewife	0.441 ± 0.627	0.09	0.48	-0.79, 2.939
Service	1.027 ± 0.464	0.182	0.027	0.115, 1.939
Business	0.75 ± 0.487	0.115	0.125	-0.209, 1.708
Monthly family income	3.394E-06	0.029	0.537	0.000, 0.000
Family history of diabetes	-0.696 ± 0.224	-0.142	0.002	-1.136, 0.256
Acquisition of information	-0.482 ± 0.348	-0.063	0.167	-1.165, 0.202
BMI	0.075 ± 0.029	0.116	0.01	0.018, 0.132
**b. Dependent variable: Attitude**				
Age	0.007 ± 0.014	-0.024	0.607	-0.034, 0.02
Sex	0.897 ± 0.574	0.152	0.119	-0.232, 2.025
Education				
Illiterate	Reference category	-	-	-
Primary	0.580 ± 0.415	0.076	0.164	-0.237, 1.396
Secondary-higher secondary	0.805 ± 0.384	0.135	0.037	0.05, 1.559
Graduate and above	0.864 ± 0.511	0.116	0.092	-0.14, 1.869
Occupation				
Unemployed	Reference category	-	-	-
Housewife	-1.44 ± 0.72	-0.246	0.047	-2.865, 0.022
Service	-0.405 ± 0.541	-0.06	0.454	-1.46, 0.658
Business	0.34 ± 0.565	0.044	0.547	-0.770,1.451
Monthly family income	6.172E-06	0.044	0.333	0.000, 0.000
Family history of diabetes	0.522 ± 0.210	0.106	0.013	0.109, 0.935
Acquisition of information	-0.512 ± 0.403	-0.056	0.204	-1.304, 0.280
BMI	0.019 ± 0.034	0.025	0.571	-0.048, 0.086
Knowledge	0.514 ± 0.058	0.43	0.0001	0.399, 0.629

*BMI* = Body Mass Index.

[1 = Unstandardized sample regression co- efficient, 2 = Standardized sample regression co- efficient].

Adjusted R^2^ (a) for Knowledge- 27%; Overall model F-test, p = 0.0001 (b) for Attitude- 31%; Overall model F-test, p = 0.0001.

## Discussion

The increasing prevalence of diabetes and its complications in Bangladesh would pose a real threat to existing health services. Awareness of the risk factors of diabetes can assist in its early prevention and reduce its incidence. Level of awareness depends on socioeconomic gradient, culture and ethnic variation [22–24]. Understanding of these variables is highly important in designing strategies for the prevention of diabetes.

Overall, the level of knowledge of the study respondents was average. The findings of the present study were quite similar to the findings of studies conducted on knowledge on risk factors of diabetes in different population groups [12, 25]. The majority of the study respondents agreed that overweight, lack of exercise and sedentary lifestyle are the risk factors of diabetes and more than half of the respondents thought that family history, oily food, fast food, and soft drinks are the risk factors of diabetes. The findings are consistent to the study conducted in Saudi Arabia, where obesity and lack of physical exercise were the risk factors of diabetes as most frequently stated by the respondents [26]. However, in a Chennai study, it was found that knowledge about the role of obesity and physical inactivity in the occurrence of diabetes was very low, with only 12% of study subjects reported these as the risk factors for diabetes [25]. In this study, many respondents were not aware of other major risk factors, such as gestational diabetes mellitus (GDM), IGT, hypercholesterolemia, hypertension, and smoking. International literature has portrayed that people with IGT are at an increased risk of type-2 diabetes, and 50% of them actually develop the disease [27]. Women with previous GDM are more likely to have modifiable risk factors for developing diabetes than women without diabetes [28]. Although GDM and IGT are two important contributors to the development of diabetes, awareness of these risk factors is very poor (26% and 3% respectively). The CURES-9 study in Chennai found that only 0.01% of people knew about smoking as a risk factor of diabetes [25]. Although in the present study, it was pretty higher (28%), creation of more awareness of the harmful effects of smoking is needed.

With regard to attitudes towards the risk factors of diabetes, majority of the respondents agreed that prevention of diabetes is possible by dietary management, nevertheless, a number of respondents thought that regular exercise needs lots of effort. Many respondents agreed that everybody should aware of prevention of diabetes with increasing age and if people try, they can reduce the risk of diabetes. However, nearly 13% of the respondents showed negative attitude, and they believed that, if there is possibility to occur diabetes in them, they have nothing to do. It was also found that an ample number of the respondents were undecided about the statements. As the respondents had common misconceptions that it can lead to a negative attitude towards the prevention of diabetes that may show poor practice of diabetes prevention.

The level of education showed significant effect on the knowledge and attitude scores which also supports findings of several studies [26, 29–32]. It was found in this study that the literate respondents had higher knowledge and attitude score than the illiterate respondents. Results of recent studies in Saudi Arabia and Oman found a similar positive association between educational status and knowledge about risk factors of diabetes [26, 29]. In the present study, a positive family history was found to be associated with the level of awareness which is similar to the findings of other studies [33–35]. Results of a study on siblings showed that having a parent with diabetes strongly and independently predicts awareness of being likely to develop diabetes [30]. Findings of another study suggest that having a family history of diabetes increases more daily consumption of fruits and vegetables and participation in diabetes screening [31]. A study conducted in Pakistan to assess the level of awareness about diabetes reported that 65% of adults with a family history of diabetes were aware of DM while only 32% of people without a positive family history were aware of the disease (p < 0.001) [32].

In this study, the mean knowledge score was higher among those who were in employment and the mean knowledge and attitude score were the lowest among housewives. In a developing country like Bangladesh, females are lagging behind compared to males in all spheres of life. It is interesting to note that the knowledge score was significantly higher among the overweight and obese group than the normal and underweight group. The study indicates that although the overweight and obese groups were aware of the risk factors of diabetes, they were not translating it to healthier lifestyles, such as following healthy diet and engaging in physical activity which is consistent with other study. A study conducted on KAP regarding obesity among Bangladeshi diabetic patients that knowledge score was almost similar between normal and obese group but practice score was the lowest in the obese group than that of others [17].

However, generalization of the results about all Bangladeshi is not possible, as this study was conducted in a single tertiary hospital with a small sample size, where people are self-motivated, the results may not be giving the true reflection of all general population. Moreover, the study conducted in a urban hospital where education may be readily accessible raises further concern as there is more likelihood that the majority of people, especially those living in rural areas and having less access to information, might have even poorer perception and practices. The authors acknowledged the small sample size and study area-tertiary hospital are two major concerns for external validation of this study. Thus, another study can be conducted on a large scale in Bangladesh to design a diabetes-awareness program to promote prevention considering the economic burden of the treatment of diabetes.

## Conclusion

Overall, the respondents participated in this study have average awareness regarding risk factors of diabetes. Diabetes education and socio-demographic factors need to be considered to improve the awareness regarding the risk factors of type-2 diabetes. Several studies [5, 6] have shown that type 2 diabetes can be prevented with the modification of lifestyles and by educating people or developing awareness about the risk factors. From public health perspective, there is a critical need for innovative target oriented prevention programs for people who are at risk of diabetes as awareness programs may motivate general population and high-risk individuals to adopt a healthy lifestyle, undergo routine medical check-ups, and be an active player in the prevention of diabetes.
